# Sulcus vocalis: probable genetic etiology. Report of four cases in close relatives

**DOI:** 10.1016/S1808-8694(15)30112-9

**Published:** 2015-10-19

**Authors:** Regina Helena Garcia Martins, Rafael Silva, Danilo Moretti Ferreira, Norimar Hernandes Dias

**Affiliations:** a Assistant Professor, PhD in Surgery - Botucatu Medical School - Unesp. Head of the Phoniatry and voice ward. Professor of Otorhinolaryngology - Universidade Estadual Paulista-Unesp, Campus de Botucatu.; b Medical student - Faculdade de Medicina de Botucatu - UNESP.; c Assistant Professor. PhD. Head of the Genetic Counseling Department - IBB (UNESP).; d MD. Otorhinolaryngologist - Department of Otorhinolaryngology - Botucatu Medical School. M.S. in Surgery (Unesp). Discipline of Otorhinolaryngology - Medical School of Botucatu (UNESP)

**Keywords:** dysphonia, aetiology, genetic, vocal sulcus

## INTRODUCTION

Sulcus vocalis is a linear depression on the mucosal cover of the vocal folds, parallel to the free border, of variable depth, usually bilateral and symmetrical^1^, ^2^. Its etiology is controversial. Bouchayer et al.^3^ consider it to be congenital because of hoarseness in childhood, its diagnosis in the larynx of children and in many family members, and the very association with other congenital laryngeal lesions. Others consider it to be acquired and secondary to inflammatory or atrophic processes on the vocal cords^2^.

The goal of the present study is to describe the presence of sulcus vocalis in four affected members of the same family, reinforcing genetic etiology.

## PRESENTATION OF THE CASES

The patients presented in this paper came from the University Hospital of the Medical School of Botucatu (Unesp).

### Case 1

**Figure 1:**
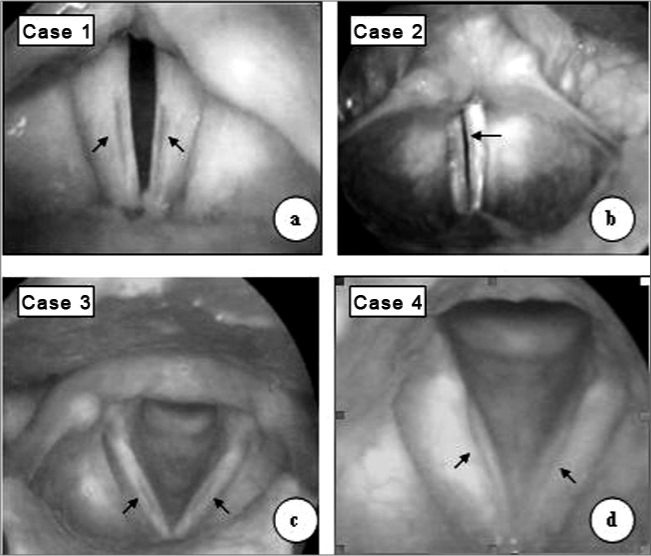
In a, c e d - bilateral sulcus vocalis (arrows); in b, sulcus vocalis to the left side (single arrow).

NAR, female, 44 years old, married, housewife, complained of hoarseness, vocal tiredness and weak voice since childhood. She did not smoke and did not complain of reflux. Her voice sounded high, blowy, with vocal effort and reduction in phonation time. With scopes (rigid 7mm, 70º - Machida telescope) we observed a sulcus vocalis in the stria major, bilaterally (Figure 1a) and a spindle-like cleft. The patient refused to undergo surgery.

The patient had four brothers, three of them had dysphonia and were called upon for vocal assessment, corresponding to the cases described hereon.

### Case 2

EAR, male, 22 years, student, single, complained of hoarseness and weak voice since childhood. He denied smoking and reported vocal abuse. His vocal characteristics were similar to the ones aforementioned, however less blowy voice. During telescopic exam we diagnosed unilateral sulcus vocalis and spindle-like cleft (Figure 1b). Phonosurgery was carried out with fat graft.

### Case 3

IGR, male, 42 years, married, receptionist, complained of hoarseness, vocal tiredness and weak voice since childhood. He denied smoking, respiratory or reflux symptoms. His voice was soft, high and blowy, with muscle efforts during voice emission. Phonation times were reduced. During telescopic exam we identified bilateral sulcus vocalis and spindle-like cleft (Figure 1c). The patient refused surgery.

### Case 4

ER, 29 years, male, single, salesman, complained of mild hoarseness during vocal abuse. He did not have respiratory or reflux symptoms and did not smoke. His voice was high and slightly blowy. During exam with the scope, we found a deep and bilateral sulcus vocalis and mild spindle-like cleft (Figure 1d), and phonosurgery was indicated.

The patients in this study did not report dysphonia in other family members. Genetic assessment did not show phenotype or karyotype alterations.

## DISCUSSION

The origin of sulcus vocalis is still controversial; some believe it to be congenital and results from a failure in the development of 4th and 6th branchial arches^3^. In these studies, symptoms are present since childhood, seen in cases 1, 2 and 3 of the present investigation. Others believe it is acquired and secondary to chronic inflammatory processes, or to atrophic processes on the vocal folds (chronic laryngitis, reflux, paralysis, smoking and presbyphonia)^1^, ^2^.

We did not see congenital lesions associated with sulcus vocalis, however, there are literature descriptions of such occurrence, especially of cysts, diagnosed in 15% of the 115 patients evaluated by Bouchayer et al.^3^

In this study, the presence of a sulcus vocalis in four members of the family reinforces the hypothesis of genetic origin, even without detecting alterations to phenotype or karyotype.

Sulcus vocalis treatment choice depends on the degree of glottic failure and vocal alterations, and include: injections of collagen, Teflon, hyaluronic acid, fat grafting and temporal muscle pre-fascia grafting; thyroplasty, mucosal slicing and sulcus ressection^4^, ^5^, ^6^. In the present investigation, only the patient from case 2 was submitted to surgery, using fat grafting.

## FINAL COMMENTS

Among the family members presented here, they had all been dysphonic since childhood, thus favoring the hypothesis of a congenital anomaly. Sulcus vocalis being present in four of the five siblings corroborates the hypothesis of genetic etiology, however inheritance pattern could not be confirmed due to the lack of laryngoscopic evaluation on the other family members.
